# Supplementary material for: Stimulator of Interferon Genes Pathway Activation through the Controlled Release of STINGel Mediates Analgesia and Anti-Cancer Effects in Oral Squamous Cell Carcinoma
Source: Biomedicines. 2024 Apr 21;12(4):920. doi: 10.3390/biomedicines12040920 (PMC11047833; doi:10.3390/biomedicines12040920)
Supplement: Supplementary file 1 [file biomedicines-12-00920-s001.zip › biomedicines-2975981-supplementary.pptx]

## Slide 1
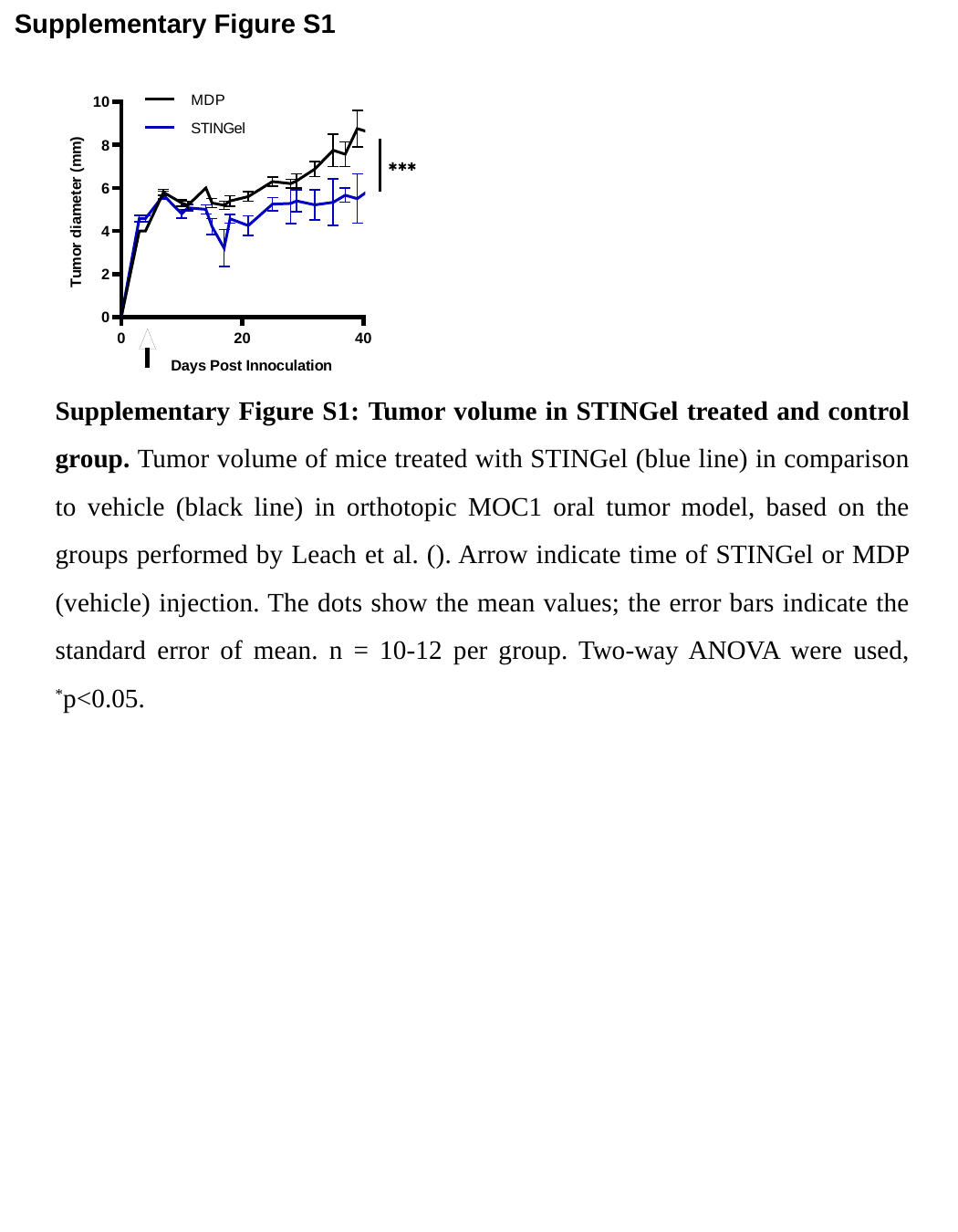

Supplementary Figure S1
Supplementary Figure S1: Tumor volume in STINGel treated and control group. Tumor volume of mice treated with STINGel (blue line) in comparison to vehicle (black line) in orthotopic MOC1 oral tumor model, based on the groups performed by Leach et al. (). Arrow indicate time of STINGel or MDP (vehicle) injection. The dots show the mean values; the error bars indicate the standard error of mean. n = 10-12 per group. Two-way ANOVA were used, *p<0.05.

## Slide 2
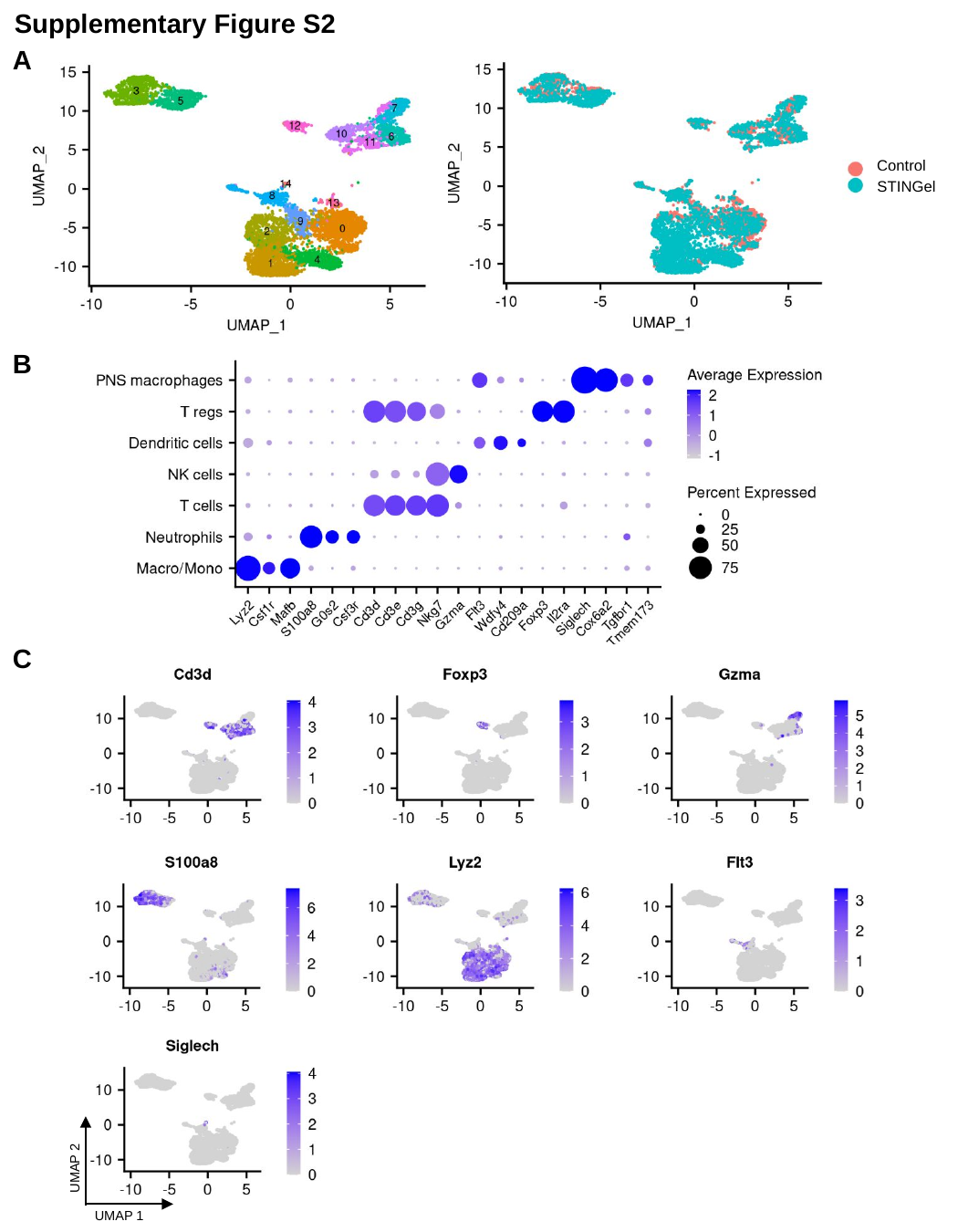

Supplementary Figure S2
A
Control
STINGel
B
C
UMAP 2
UMAP 1

## Slide 3
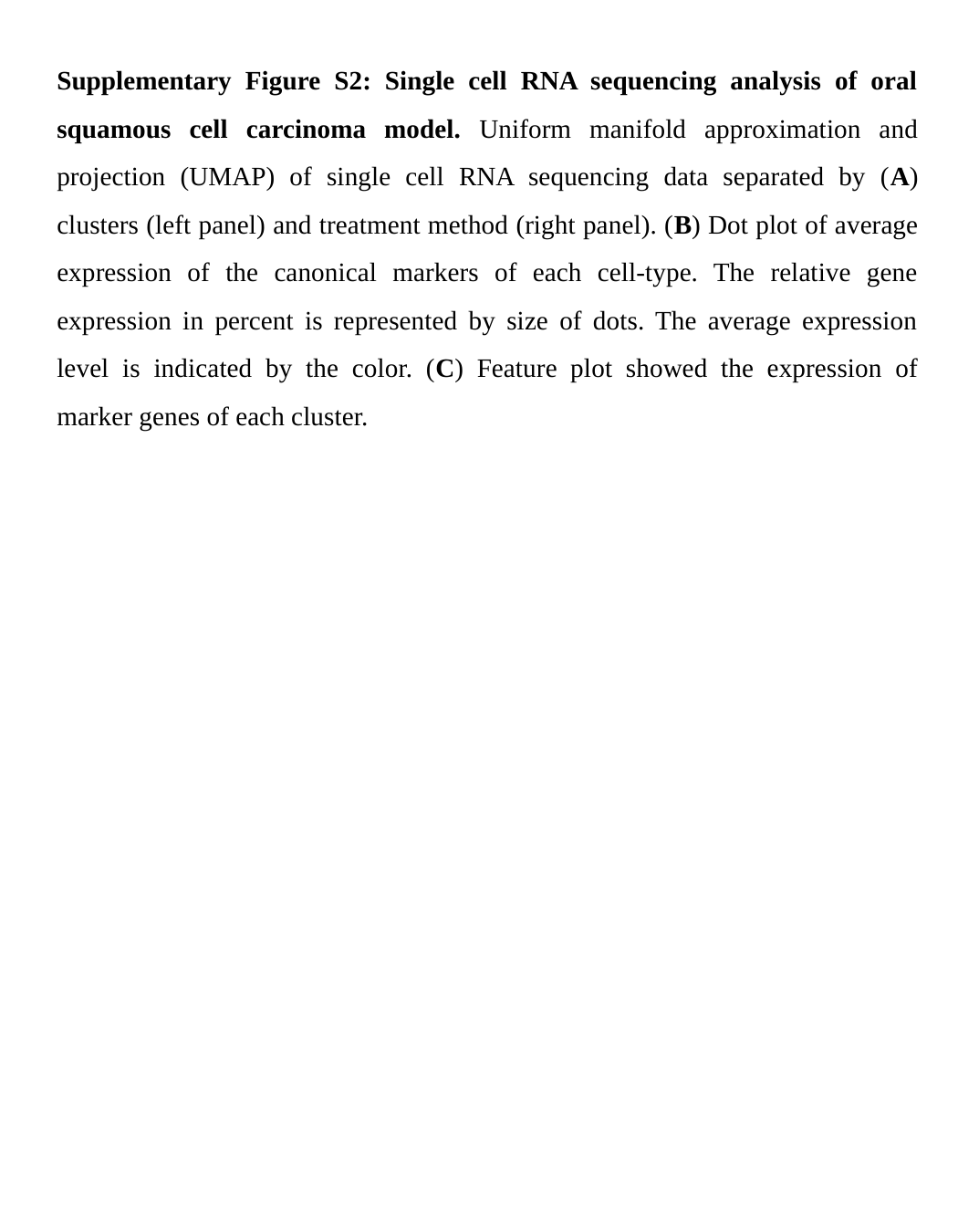

# Supplementary Figure S2: Single cell RNA sequencing analysis of oral squamous cell carcinoma model. Uniform manifold approximation and projection (UMAP) of single cell RNA sequencing data separated by (A) clusters (left panel) and treatment method (right panel). (B) Dot plot of average expression of the canonical markers of each cell-type. The relative gene expression in percent is represented by size of dots. The average expression level is indicated by the color. (C) Feature plot showed the expression of marker genes of each cluster.

## Slide 4
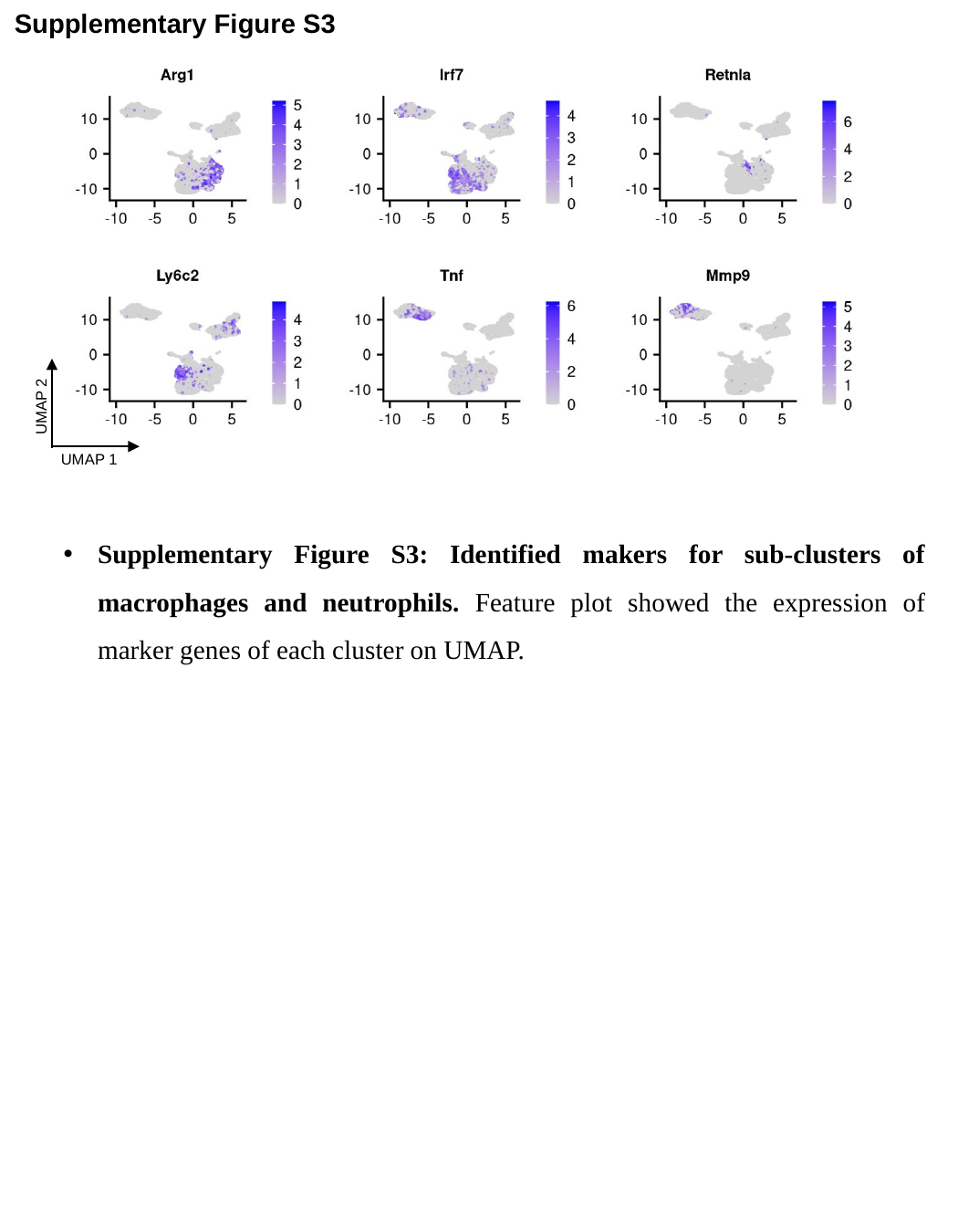

Supplementary Figure S3
UMAP 2
UMAP 1
# Supplementary Figure S3: Identified makers for sub-clusters of macrophages and neutrophils. Feature plot showed the expression of marker genes of each cluster on UMAP.

## Slide 5
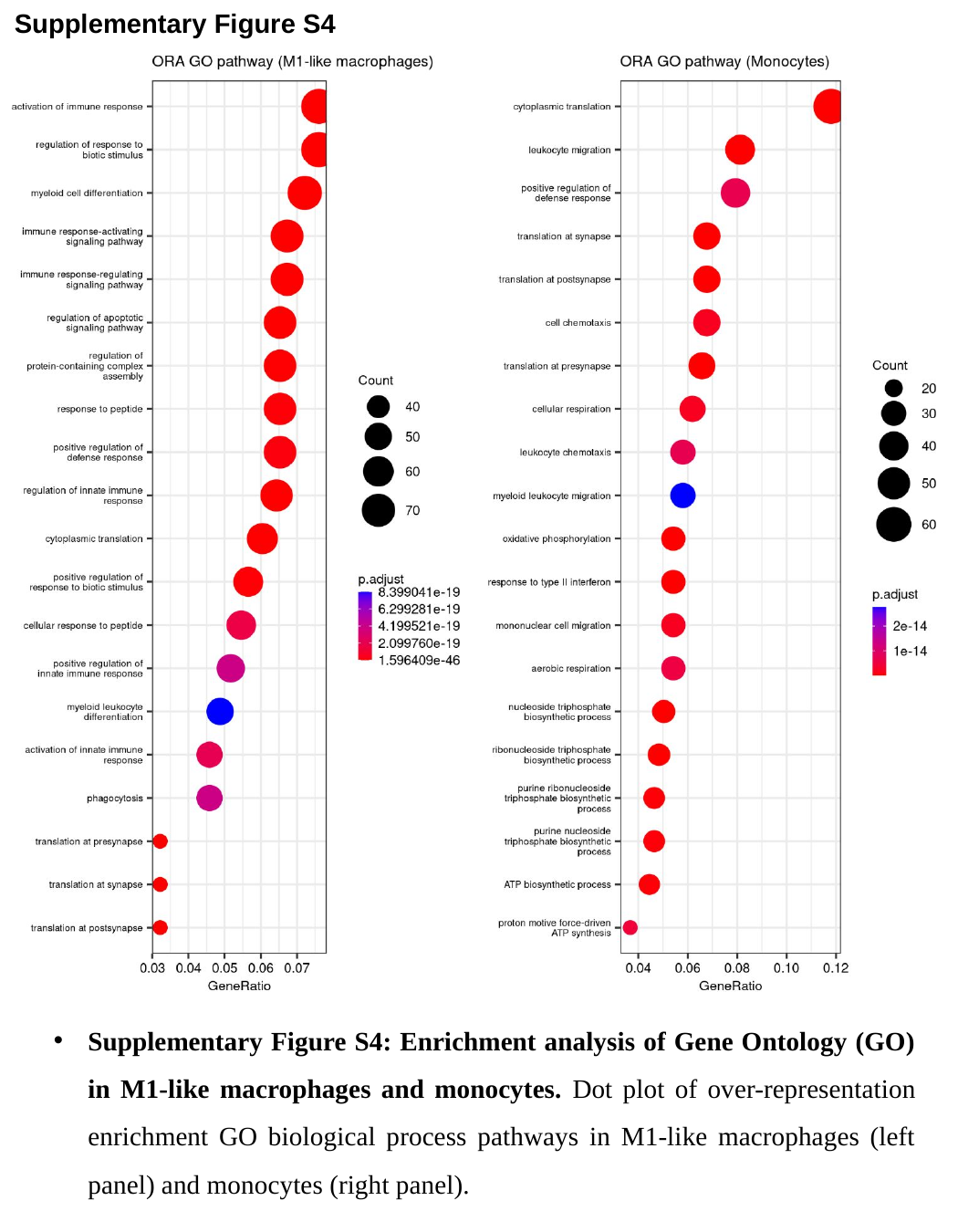

Supplementary Figure S4
# Supplementary Figure S4: Enrichment analysis of Gene Ontology (GO) in M1-like macrophages and monocytes. Dot plot of over-representation enrichment GO biological process pathways in M1-like macrophages (left panel) and monocytes (right panel).

## Slide 6
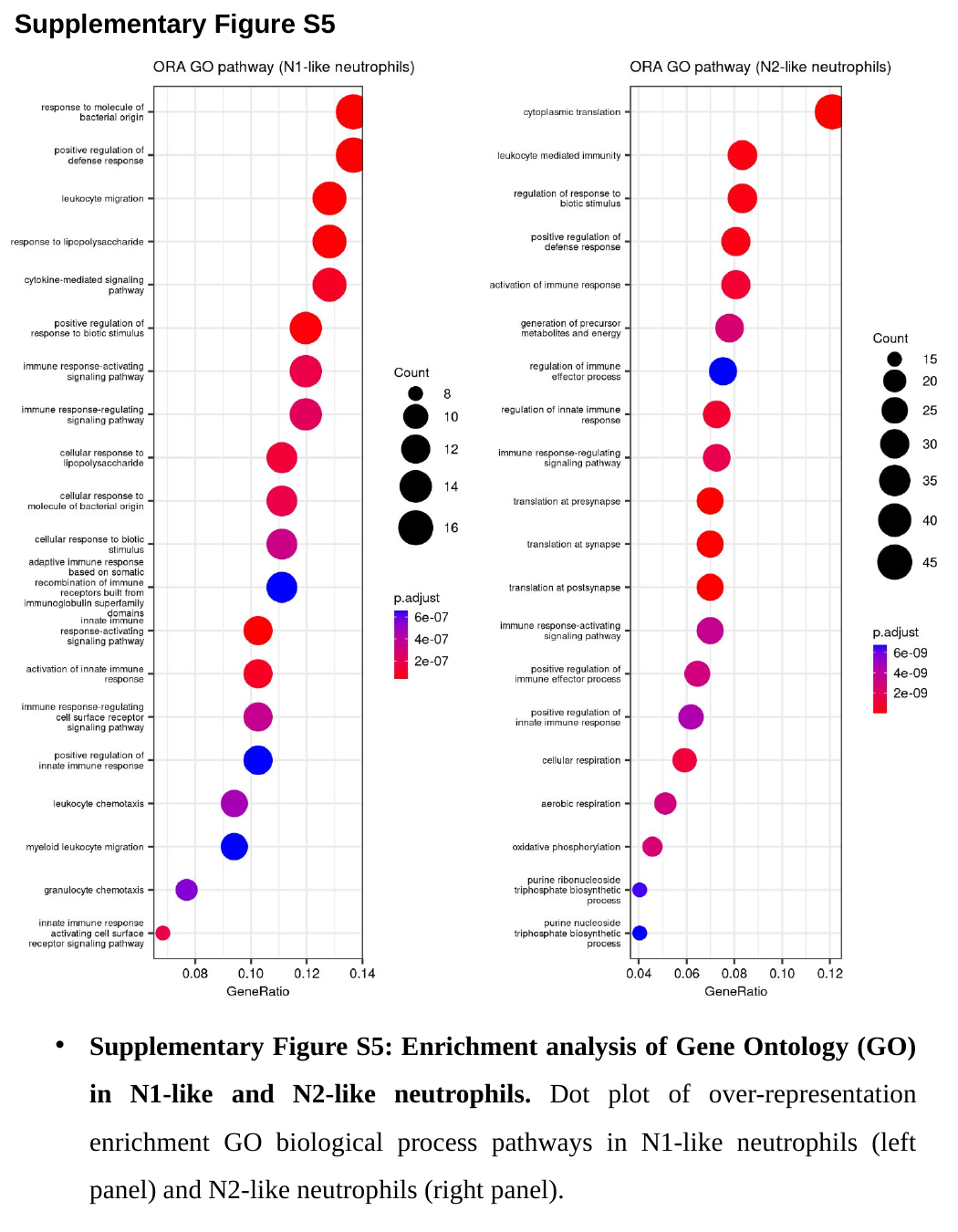

Supplementary Figure S5
# Supplementary Figure S5: Enrichment analysis of Gene Ontology (GO) in N1-like and N2-like neutrophils. Dot plot of over-representation enrichment GO biological process pathways in N1-like neutrophils (left panel) and N2-like neutrophils (right panel).

## Slide 7
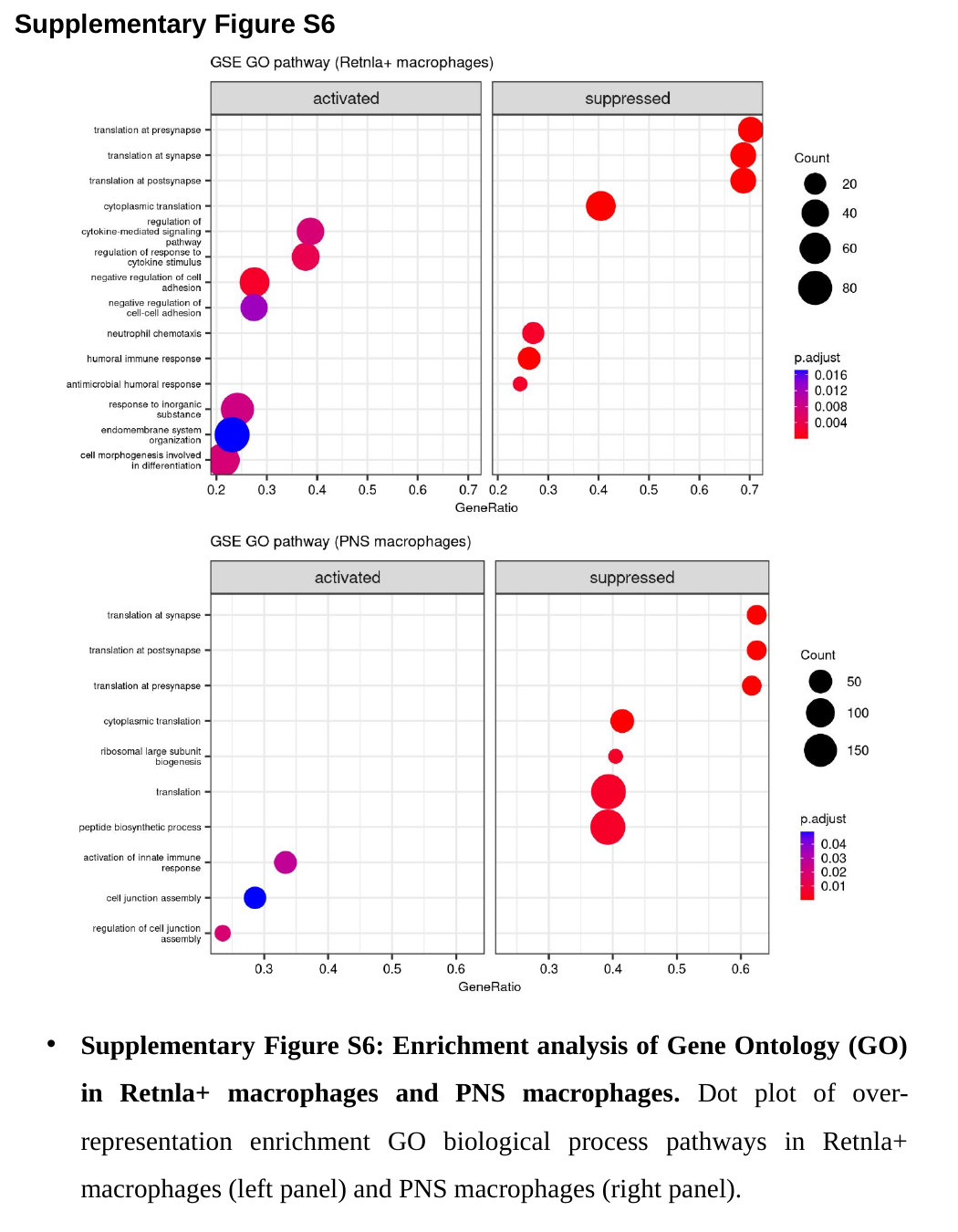

Supplementary Figure S6
# Supplementary Figure S6: Enrichment analysis of Gene Ontology (GO) in Retnla+ macrophages and PNS macrophages. Dot plot of over-representation enrichment GO biological process pathways in Retnla+ macrophages (left panel) and PNS macrophages (right panel).

## Slide 8
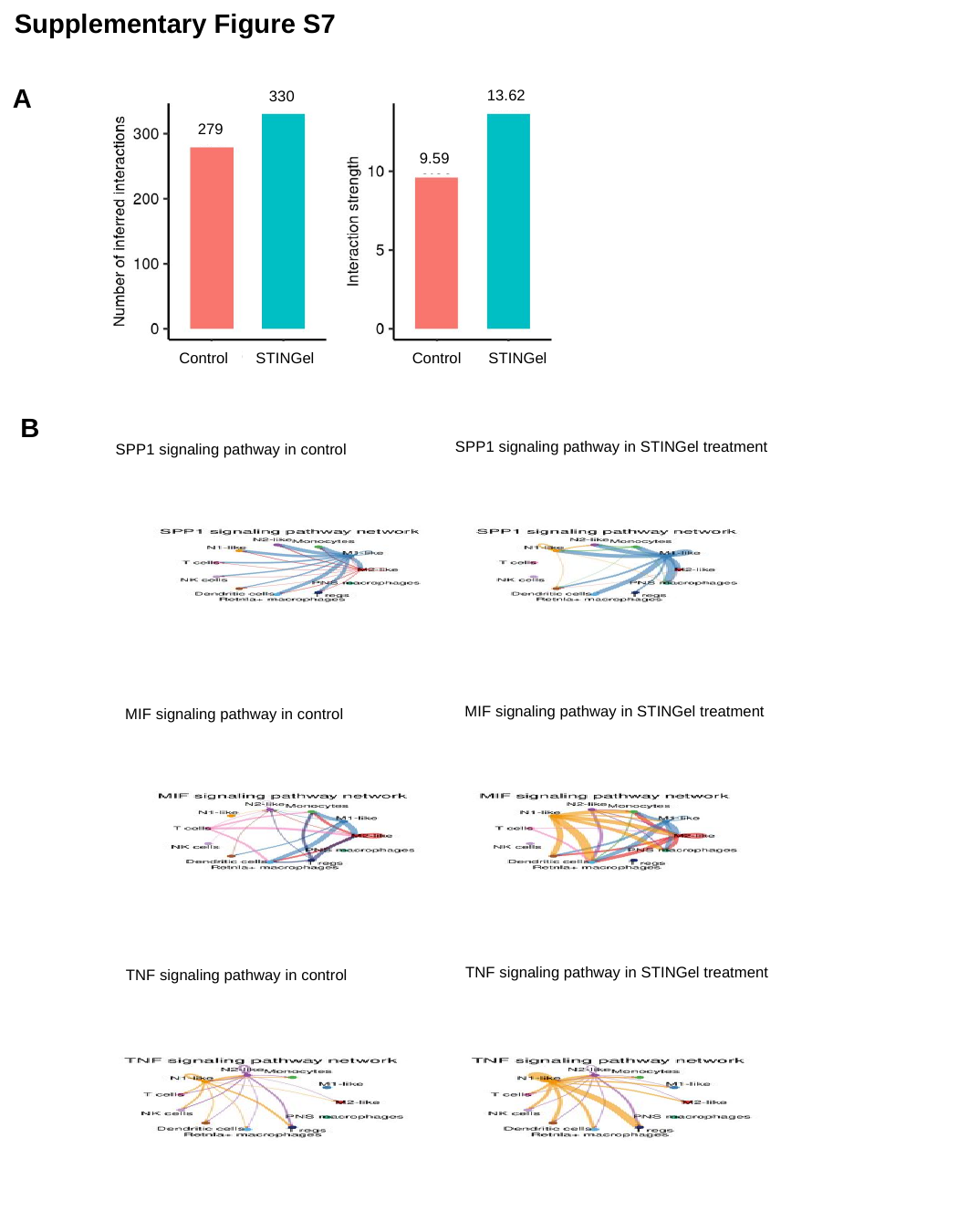

Supplementary Figure S7
A
13.62
330
279
9.59
Control
STINGel
Control
STINGel
B
SPP1 signaling pathway in STINGel treatment
SPP1 signaling pathway in control
MIF signaling pathway in STINGel treatment
MIF signaling pathway in control
TNF signaling pathway in STINGel treatment
TNF signaling pathway in control

## Slide 9
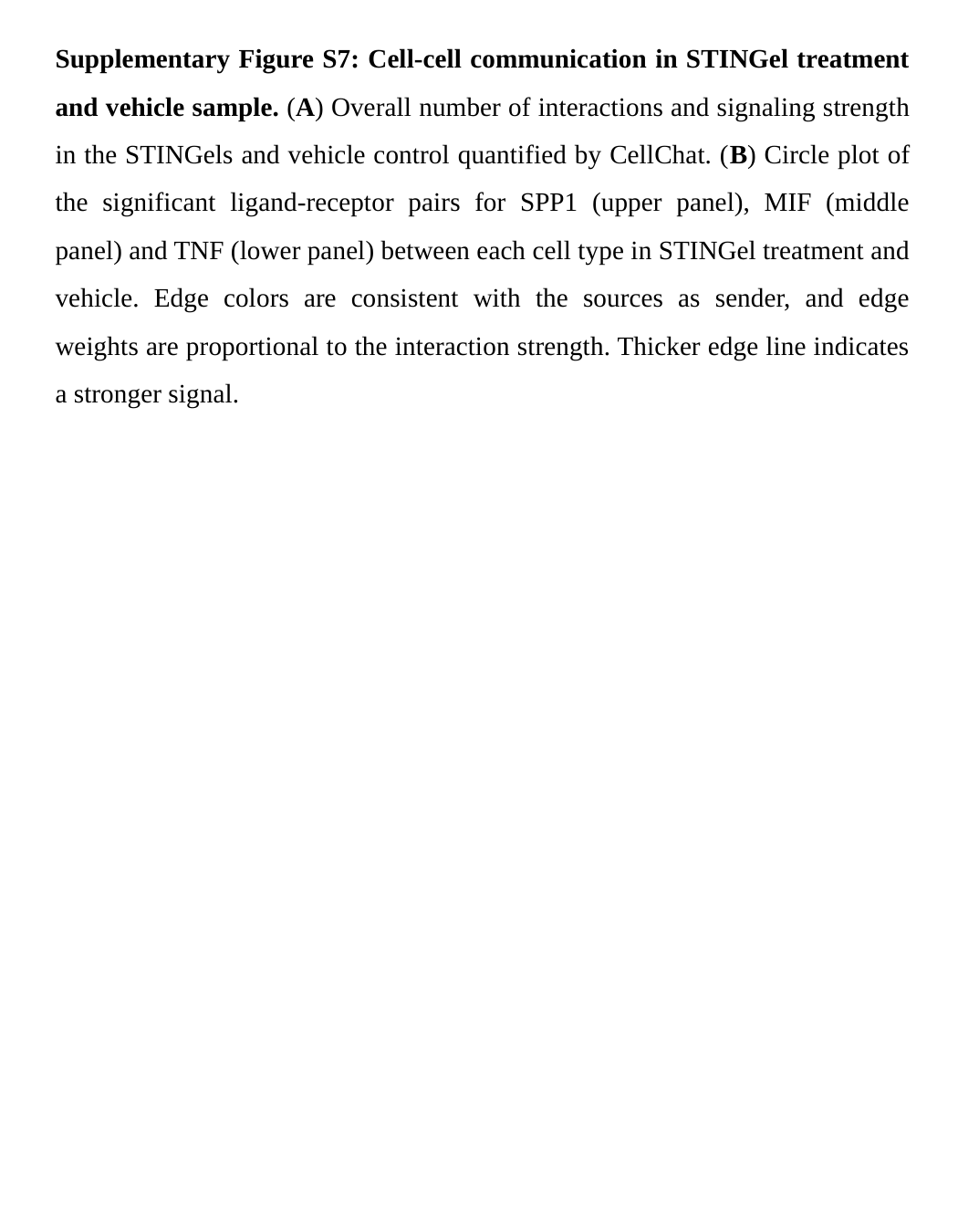

Supplementary Figure S7: Cell-cell communication in STINGel treatment and vehicle sample. (A) Overall number of interactions and signaling strength in the STINGels and vehicle control quantified by CellChat. (B) Circle plot of the significant ligand-receptor pairs for SPP1 (upper panel), MIF (middle panel) and TNF (lower panel) between each cell type in STINGel treatment and vehicle. Edge colors are consistent with the sources as sender, and edge weights are proportional to the interaction strength. Thicker edge line indicates a stronger signal.
